# Altered Serum Amino Acid and Acylcarnitine Profiles in Hyperinsulinemic Hypoglycemia and Ketotic Hypoglycemia

**DOI:** 10.3389/fendo.2020.577373

**Published:** 2020-10-08

**Authors:** Zhen-Ran Xu, Xiao-Yi Zhu, Wei Lu, Wei-Hua Sun, Ruo-Qian Cheng, Jin-Wen Ni, Li Xi, Khalid Hussain, Fei-Hong Luo, Miao-Ying Zhang

**Affiliations:** ^1^Department of Pediatric Endocrinology and Inherited Metabolic Diseases, Children’s Hospital of Fudan University, Shanghai, China; ^2^Pediatric Research Institute, Children's Hospital of Fudan University, Shanghai, China; ^3^Department of Pediatric Medicine, Weill Cornell Medicine, Division of Endocrinology, Doha, Qatar

**Keywords:** hypoglycemia, hyperinsulinemic hypoglycemia, amino acid, acylcarnitine, ketotic hypoglycemia

## Abstract

**Background:**

In addition to inborn metabolic disorders, altered metabolic profiles were reported to be associated with the risk and prognosis of some non-metabolic diseases, while as a rare metabolic disease, the overall secondary metabolic spectrum in congenital hyperinsulinemic hypoglycemia (HH) is largely undetermined. Therefore, we investigated metabolic profiles in HH patients and used ketotic hypoglycemia (KH) patients as a control cohort to unveil their distinct metabolic features.

**Methods:**

A total of 97 hypoglycemia children, including 74 with hyperinsulinemic hypoglycemia and 23 with ketotic hypoglycemia, and 170 euglycemia control subjects were studied retrospectively. Clinical and biochemical data were collected. The normoglycemic spectra of amino acids and acylcarnitines were determined by liquid chromatography tandem mass spectrometry. The serum insulin and fatty acid concentrations during standardized fasting tests in hypoglycemia patients were also collected. Receiver operating characteristic curve analysis was performed to screen potential biomarkers.

**Results:**

Among the normoglycemic spectra of amino acids, blood valine (*p* < 0.001), arginine (*p* < 0.001), threonine (*p* = 0.001), glutamate (*p* = 0.002), methionine (*p* = 0.005), ornithine (*p* = 0.008), leucine (*p* = 0.014), alanine (*p* = 0.017), proline (*p* = 0.031), citrulline (*p* = 0.042), aspartate (*p* = 0.046), and glycine (*p* = 0.048) levels differed significantly among the three groups. Significantly decreased levels of long- (C14:1, *p* < 0.001; C18, *p* < 0.001), medium- (C8, *p* < 0.001; C10, *p* < 0.001; C10:1, *p* < 0.001), and short-chain (C4-OH, *p* < 0.001; C5OH, *p* < 0.001) acylcarnitines were found in the hyperinsulinemic hypoglycemia group. Hyperinsulinemic hypoglycemia children with focal lesions and diffuse lesions had similar amino acid and acylcarnitine spectra. C10:1 < 0.09 μmol/L, threonine > 35 μmol/L, and threonine/C10:1 > 440 showed sensitivities of 81.1, 66.2, and 81.1% and specificities of 72.7, 78.3, and 81.8%, respectively, in distinguishing HH from KH.

**Conclusions:**

We found significantly different altered serum amino acid and acylcarnitine profiles at normoglycemia, especially decreased C10:1 and increased threonine levels, between HH and KH children, which may reflect the insulin ketogenesis inhibition effect in HH patients; however, the detailed mechanisms and physiological roles remain to be studied in the future.

## Introduction

Hypoglycemia is a common pediatric emergency, and serious hypoglycemia might cause pediatric encephalopathy and intellectual impairment (1, 2). During the neonatal and infant periods, hyperinsulinemic hypoglycemia (HH) is the most common etiology of refractory hypoglycemia resulting from congenital gene mutations (3, 4), while ketotic hypoglycemia (KH), which is characterized by high ketone body concentrations, is more common in patients older than 1 year of age (5) despite the small portion of inborn early-onset ketone-related metabolic disorders (6). Children with HH and KH have different treatments and prognoses (7–9).

Insulin is the key hormone for maintaining glucose homeostasis and regulates metabolism in several aspects. Under normal physiological conditions, insulin suppresses counter-regulatory responses, including gluconeogenesis, proteolysis, lipolysis, fatty acid oxidation and ketogenesis, and increases the uptake of glucose from peripheral tissues (10). However, HH patients experience dysregulated insulin secretion, which leads to recurrent hypoglycemia. Therefore, HH children have different metabolic profiles from normal children and children with KH.

Except for the screening of inborn metabolic disorders, the spectra of amino acids and acylcarnitines were also reported to be associated with the risk and prognosis of some non-metabolic diseases (11–13). Previous studies have shown suppressed lipolysis and ketogenesis in HH children based on decreased serum concentrations of β-hydroxybutyrate and free fatty acids (FFAs), which may also serve as indicators for the diagnosis of HH (14, 15). However, the detailed amino acid and fat metabolic profiles of HH are still unknown. Nearly four decades ago, altered serum branched-chain amino acid levels in 14 children with documented KH and in six infants with documented hyperinsulinism were reported (16). However, no further study has been reported regarding the full spectra of acylcarnitine and amino acid metabolic profiles in patients with HH or KH.

We hypothesized that the pathophysiological characteristics of HH would elicit unique secondary metabolic profile changes in HH patients rather than merely a reduction in FFAs. Therefore, we aimed to identify differences in metabolic profiles between HH and KH to reveal the possible pathophysiology and to explore their diagnostic value.

## Materials and Methods

### Subjects

We retrospectively reviewed all cases with a diagnosis of hypoglycemia in our center from 2011 to 2018 according to the computerized medical record system. In total, we identified 74 children diagnosed with HH and 23 children diagnosed with KH. HH and KH were diagnosed based on the pediatric endocrinologists’ chart notes (15, 17). Children with glycogen storage diseases, pituitary dysplasia, adrenal disorders, organic acid metabolism disorder, and other inherited metabolic diseases were excluded. A flow chart is shown in **Supplementary Figure 1**.

For HH cases, we examined the charts for evidence to validate the diagnosis. The following parameters were used to validate the diagnosis: elevated serum concentrations of insulin and C-peptide when blood glucose levels were <2.6 mmol/L; reduced serum levels of β-hydroxybutyrate, acetoacetic acid and FFAs; a glucose infusion rate >8 mg/kg/min to maintain euglycemia; a blood glucose increase >1.5 mmol/L after an injection of glucagon; and positive ^18^F-DOPA PET scan results (17). Due to domestic technical unavailability, 50 of the 74 HH patients received ^18^F-DOPA PET scan to confirm pancreas lesions starting in 2017 (18–20). For KH cases, we also examined charts for evidence of elevated urine ketones or serum β-hydroxybutyrate at the time of hypoglycemia to validate the diagnosis. We also reviewed medical histories to exclude children with diabetes, genetic syndromes, or other metabolic defects in gluconeogenesis, glycogenolysis, proteolysis, lipolysis, fatty acid oxidation, or ketogenesis (15). We included only KH children in the acute phase who experienced hypoglycemia within one week before admission. Data, including age, sex, birthweight, and normoglycemic spectra of amino acids and acylcarnitines, were collected. Ethical permission was received from the Ethics Commission of Children’s Hospital of Fudan University (No. 2015-154).

To compare the normoglycemic spectra of amino acids and acylcarnitines in HH and KH children with those of normal children, we randomly selected 170 cases from the hospital’s Tandem Mass Laboratory’s normal reference database. The reference subjects did not have any metabolic disorder, liver dysfunction, gastrointestinal disorder, malnutrition, or other known disorder (21).

### Liquid Chromatography Tandem Mass Spectrometry Assay

For amino acid and acylcarnitine spectrometry measurements, finger blood drops were collected on filter paper (S&S 903, Bio-Rad, USA) at normoglycemic status. The spectra of amino acids and acylcarnitines were assayed by liquid chromatography tandem mass spectrometry (LC-MS/MS, H ClassXevo TQD, Waters, USA). Dot blood samples provided by the Centers for Disease Control of the United States served as quality control. All tests were performed in the central laboratory of the hospital. The tests for amino acid and acylcarnitine spectrometry were completed within 48 h of sampling.

### Fasting Test for Hyperglycemia Subjects

According to the medical records, the fasting test was performed for 74 cases of HH and 18 cases of KH, and the other cases denied the fasting test. The fasting tests were performed according to standard procedures (22, 23). Octreotide, glucagon, and diazoxide were stopped one day before the fasting test. The fasting programs were adjusted according to the children’s condition, including fasting until the blood glucose level was <2.6 mmol/L or weaning of the intravenous glucose infusion rate by decreases of 2 mg/kg/min every hour until the blood glucose level was <2.6 mmol/L. Bedside capillary glucose and ketone concentrations were closely monitored. The fasting test was stopped when blood glucose levels were <2.6 mmol/L or patients had suspicious hypoglycemia symptoms (24). The fasting test lasted a maximum of 12 h, and the fasting test was discontinued even if hypoglycemia did not develop. Serum concentrations of insulin and FFAs were tested at the end of the fasting test. Serum insulin levels at the end of the fasting tests were determined by chemiluminescence (IMMULITE 1000, Siemens, Germany) with a lower limit of detection of 0.03 mIU/L.

### Statistical Analysis

Data were described as numbers (proportions) for categorical variables and the means (SDs) or the medians (IQRs) for continuous variables. We used χ² tests to compare categorical variables. For normally distributed continuous variables, Student’s t test was used to compare differences between two groups, and a one-way ANOVA, including the Student-Newman-Keuls test, was used to compare significant differences among the three groups. The Mann-Whitney U test and Kruskal-Wallis test were used if variables did not conform to a normal distribution, and a Dunn-Bonferroni test was used for *post hoc* comparisons. We generated receiver operating characteristic (ROC) curves for all amino acids and acylcarnitines with significant differences between the HH group and the KH group. The corresponding area under the curve (AUC) and 95% confidence interval (95% CI) were calculated, and the cutoff value was determined using the Youden index. A *p* value <0.05 was considered statistically significant. Statistical analysis was performed using SPSS 21.0 (IBM, USA).

To validate the differences among the HH, KH and control groups, we compared the spectra of amino acids and acylcarnitines not only in all cases (74 cases of HH and 23 cases of KH) but also in the subgroups that developed hypoglycemia in the fasting test (73 cases of HH and 14 cases of KH). The sensitivity and specificity of insulin were calculated only among those who developed hypoglycemia in the fasting test (14 cases of KH and 73 cases of HH). The sensitivity and specificity of amino acids and acylcarnitines were calculated among the KH (n = 23) and HH (n = 74) cases as well as among those who developed hypoglycemia in the fasting test (14 cases of KH and 73 cases of HH).

## Results

### Basic Clinical and Biological Characteristics

Among the 74 patients with HH, 45 boys (60.8%) were included. The HH group exhibited a significantly lower mean onset age than the KH group (1.10 ± 1.80 *vs* 2.56 ± 1.79 years, *p* = 0.001) (**Table 1**). HH patients also had a higher birth weight (3.78 ± 0.78 *vs* 2.83 ± 0.88 kg, *p* < 0.001), and 50 patients with HH underwent ^18^F-DOPA PET scans. Among these HH patients, 17 (34%) showed focal lesions, while 33 patients (66%) showed diffuse lesions.

**Table 1:** Basic characteristics of hyperinsulinemic hypoglycemia and ketotic hyperglycemia patients.

	Hyperinsulinemic hypoglycemia (n = 74)	Ketotic hypoglycemia (n = 23)	*P* value
Sex			0.714
Male	45/74 (60.8%)	13/23 (56.5%)	
Female	29/74 (39.2%)	10/23 (43.5%)	
Age (years)	1.10 ± 1.80	2.56 ± 1.79	0.001
Birth weight (kg)	3.78 ± 0.78	2.83 ± 0.88	<0.001
Fasting tests			
Occurring hypoglycemia	73/74 (98.6%)	14/18 (77.8%)	<0.001
Serum glucose (mmol/L)	2.19 ± 0.69	2.31 ± 0.35	0.551
Serum insulin (mIU/L)	7.37 (3.10–18.25)	1.70 (0.53–2.95)	<0.001
Free fatty acids (μmol/L)	233.0 (138.5–438.5)	579.0 (327.8–913.3)	0.005

In total, 74 patients in the HH group and 18 in the KH group underwent fasting tests. Among these patients, 73 patients in the HH group (98.6%) and 14 patients in the KH group (77.8%) developed hypoglycemia within 12 h. The HH group had significantly higher serum insulin levels during hypoglycemia compared to the KH group (*p* < 0.001). When applying a cutoff value of insulin of 1 mIU/L, the sensitivity was 93.2% (68 of 73 cases), while the specificity was only 42.9% (6 of 14 cases). In addition, serum FFA levels were significantly decreased in the HH group (*p* = 0.005) compared to the KH group.

### Amino Acid Spectra

**Figure 1:**
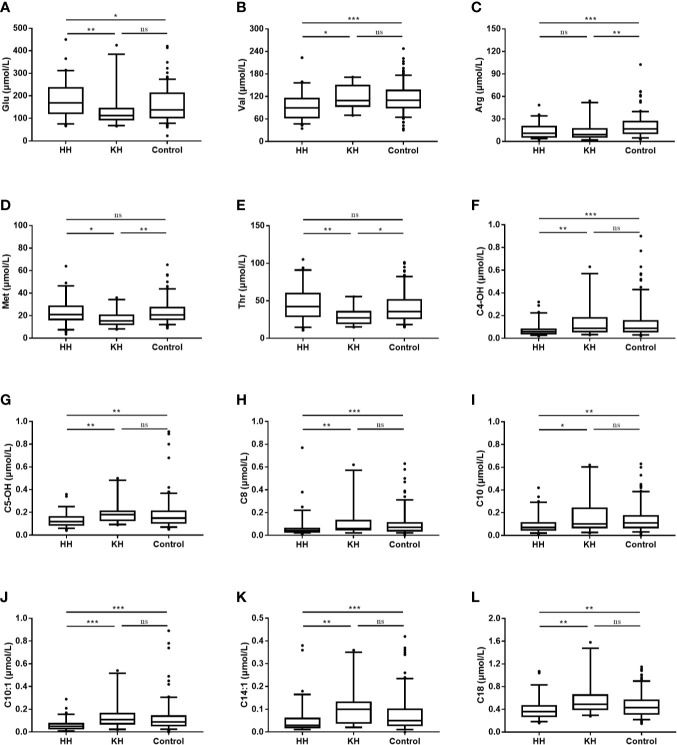
Differences in amino acid and acylcarnitine concentrations at normoglycemia among the ketotic hypoglycemia, hyperinsulinemic hypoglycemia and control groups. The box plots show the median (horizontal line), interquartile range (box), 5th percentiles (whiskers), 95th percentiles (whiskers), and outliers outside the 5th and 95th percentiles (dots). *p < 0.05 when the two groups were compared. **p < 0.01 when the two groups were compared. ***p < 0.001 when the two groups were compared. NS, Not significant; HH, hyperinsulinemic hypoglycemia; KH, ketotic hypoglycemia. **(A)** Blood glucsoce, **(B)** Valine, **(C)** Arginine, **(D)** Methionine, **(E)** Threonine, **(F)** Hydroxybutyrylcarnitine, **(G)** 3-Hydroxyisovalerylcarnitine/2-methyl-3-hydroxybutyrylcarnitine, **(H)** Octanoylcarnitine, **(I)** Decanoylcarnitine, **(J)** Decenoylcarnitine, **(K)** Tetradecenoylcarnitine, **(L)** Octadecanoylcarnitine.

Several differences in amino acid levels were found among the HH group, KH group and control group at normoglycemia, including the levels of valine (Val, *p* < 0.001), arginine (Arg, *p* < 0.001), threonine (Thr, *p* = 0.001), glutamate (Glu, *p* = 0.002), methionine (Met, *p* = 0.005), ornithine (Orn, *p* = 0.008), leucine (Leu, *p* = 0.014), alanine (Ala, *p* = 0.017), proline (Pro, p = 0.031), citrulline (Cit, *p* = 0.042), aspartate (Asp, *p* = 0.046), and glycine (Gly, *p* = 0.048) (**Table 2** and **Supplementary Table 1**). To identify potential biomarkers, we further compared the amino acids in pairs. Among the three groups, the HH group had the highest level of Glu and the lowest level of Val (a branched-chain amino acid, BCAA) (**Figure 1**). The HH group also had decreased levels of Leu, Pro, and Asp compared to the control group, but no significant differences were found between the HH group and KH group. The Arg level decreased in both the HH group and KH group compared to the control group. The concentrations of Met and Thr (two essential amino acids) as well as Ala were decreased in the KH group compared to the other groups, and no significant differences in these three amino acids were observed between the HH group and the control group.

**Table 2:** Differences in amino acid and acylcarnitine concentrations at normoglycemia among the ketotic hypoglycemia, hyperinsulinemic hypoglycemia, and control groups.

	Ketotic hypoglycemia (n = 23)	Hyperinsulinemic hypoglycemia (n = 74)	Control (n = 170)	Significant pairwise comparisons		
				HH vs Con	KH vs Con	HH vs KH
Male/Femal	13/10	45/29	96/74	NS	NS	NS
Age	2.56 ± 1.79	1.10 ± 1.81	1.67 ± 2.30	NS	NS	**
Amino acids^#^				–	–	–
Val	109.5 (91.0–149.9)	89.5 (63.2–115.7)	109.8 (90.6–135.3)	*	NS	***
Arg	9.2 (6.2–15.9)	11.0 (6.3–19.7)	16.9 (11.2–26.3)	***	**	NS
Thr	27.5 (20.5–34.0)	42.2 (29.3–59.6)	35.8 (26.6–51.3)	NS	*	**
Glu	112.0 (95.2–144.1)	168.3 (123.0–235.1)	138.3 (103.9–211.5)	*	NS	**
Met	15.4 (12.3–19.8)	20.9 (16.8–28.3)	20.5 (16.7–27.1)	NS	**	*
Orn	15.9 (12.5–21.5)	19.9 (13.6–25.6)	21.9 (17.8–28.9)	NS	*	NS
Leu	83.8 (72.3–117.6)	82.8 (63.2–100.1)	91.3 (77.7–113.5)	*	NS	NS
Ala	154.9 (117.0–219.6)	229.9 (162.1–336.3)	192.1 (145.8–267.4)	NS	NS	*
Pro	398.4 (287.0–564.4)	367.1 (212.6–578.0)	448.9 (347.0–660.3)	*	NS	NS
Cit	16.3 (11.9–21.2)	15.7 (11.9–20.5)	17.7 (13.6–22.7)	NS	NS	NS
Asp	23.5 (17.7–28.1)	21.6 (16.6–31.2)	26.5 (20.1–34.7)	*	NS	NS
Gly	154.9 (122.3–178.1)	179.2 (157.5–229.0)	170.0 (145.0–202.8)	NS	NS	NS
Acylcarnitines^#^						
C4-OH	0.08 (0.05–0.13)	0.05 (0.04–0.08)	0.07 (0.05–0.13)	***	NS	**
C5-OH	0.18 (0.14–0.19)	0.12 (0.09–0.15)	0.14 (0.10–0.18)	**	NS	**
C8	0.05 (0.04–0.06)	0.04 (0.03–0.05)	0.06 (0.04–0.09)	***	NS	**
C10	0.09 (0.06–0.10)	0.07 (0.04–0.10)	0.09 (0.06–0.14)	**	NS	*
C10:1	0.10 (0.05–0.12)	0.04 (0.03–0.07)	0.08 (0.05-0.12)	***	NS	***
C14:1	0.05 (0.03–0.10)	0.03 (0.02–0.05)	0.05 (0.03–0.07)	***	NS	**
C18	0.46 (0.40–0.51)	0.35 (0.26–0.43)	0.40 (0.32–0.51)	**	NS	**
C6	0.04 (0.03–0.05)	0.04 (0.03–0.05)	0.05 (0.03–0.06)	**	NS	NS
C10:2	0.02 (0.01–0.02)	0.01 (0.01–0.02)	0.02 (0.01–0.03)	**	NS	NS
C4	0.14 (0.11–0.18)	0.13 (0.10–0.13)	0.15 (0.12–0.19)	**	NS	NS
C12	0.06 (0.04–0.07)	0.06 (0.04–0.09)	0.08 (0.05–0.11)	**	NS	NS

^#^μmol/L. Data were represented as median (IQR). Factors were ranged from small to large in order of p value. HH, Hyperinsulinemic hypoglycemia; KH, Ketotic hypoglycemia; Con, Control; NS, Not significant. *p < 0.05 when the two groups were compared. **p < 0.01 when the two groups were compared. ***p < 0.001 when the two groups were compared.

To verify our findings, we also compared the normoglycemic amino acid spectra between KH (n = 14) and HH (n = 73) children who developed hypoglycemia in the fasting test, and the results were similar to the above findings (**Supplementary Table 2**).

### Acylcarnitine Spectra

Overall, the HH group showed a decreasing trend of acylcarnitine levels at normoglycemia (**Table 2** and **Supplementary Table 3**). Compared to those in the KH and control groups, the levels of long- (C14:1, *p* < 0.001; C18, *p* < 0.001), medium- (C8, *p* < 0.001; C10, *p* < 0.001; C10:1, *p* < 0.001), and short-chain (C4-OH, *p* < 0.001; C5OH, *p* < 0.001) acylcarnitines were significantly decreased in the HH group, while no significant differences in the acylcarnitine spectra were found between the KH group and control group (**Figure 1**). Furthermore, the HH group had lower levels of C4 (*p* = 0.007), C6 (*p* = 0.002), C10:2 (*p* = 0.002), and C12 (*p* = 0.008) than the control group, but no significant difference was observed compared to the KH group. Furthermore, we compared only the patients who developed hypoglycemia in the fasting test, and similar results were obtained (**Supplementary Table 4**).

### Metabolic Profiles of Different Lesion Types of HH

To identify whether different types of HH cause different metabolic statuses, we compared the spectra of amino acids and acylcarnitines between HH patients with focal lesions and HH patients with diffuse lesions. No significant differences were found in the levels of amino acids and acylcarnitines, except for C5-OH (*p* = 0.045). Patients with diffuse lesions had slightly higher C5-OH levels than patients with focal lesions [0.12 μmol/L (0.10–0.16) *vs* 0.09 μmol/L (0.08–0.14)].

### ROC Analysis

**Figure 2:**
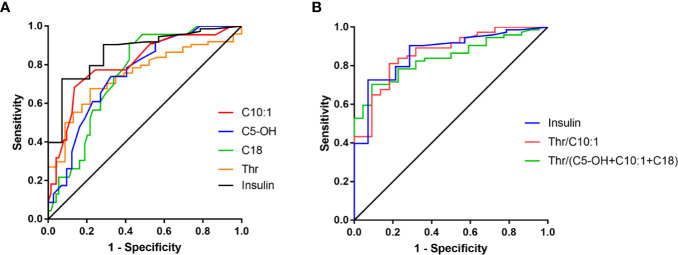
ROC curves of amino acids and acylcarnitines **(A)** and their combinations **(B)**.

According to the ROC curves, Thr, C10:1, C5-OH, C18, and C14:1 at normoglycemia each had an AUC > 0.7 with *p* < 0.001 (**Table 3** and **Figure 2**). Among these, C10:1 had the largest AUC (0.807, 95% CI: 0.698–0.915), and the threshold of C10:1, as a biomarker for the differential diagnosis of HH, was 0.09 µmol/L based on the Youden index analysis. When C10:1 < 0.09 µmol/L was used as the new screening criterion, the diagnostic specificity was substantially increased compared to that of the insulin >1.0 uIU/ml criterion (sensitivity 81.1%, specificity 72.7%). The insulin level at the time of hypoglycemia had the highest AUC of 0.866 (95% CI 0.771–0.962, *p* < 0.001) compared to the amino acid and acylcarnitine levels.

**Table 3:** Results of the ROC curve analysis of insulin, amino acids, and acylcarnitines.

	AUC	*P* value	95%CI
Insulin at the time of hypoglycemia	0.866	<0.001	0.771–0.962
Fasting free fatty acids at the time of hypoglycemia	0.716	0.005	0.577–0.855
Amino acids			
Thr	0.746	<0.001	0.643–0.850
Glu	0.739	0.001	0.623–0.856
Val	0.687	0.007	0.575–0.799
Met	0.679	0.010	0.559–0.799
Ala	0.663	0.019	0.543–0.783
Acylcarnitines			
C10:1	0.807	<0.001	0.698–0.915
C5-OH	0.753	<0.001	0.648–0.858
C18	0.752	<0.001	0.652–0.851
C14:1	0.741	<0.001	0.628–0.855
C4-OH	0.714	0.002	0.598–0.831
C8	0.709	0.002	0.585–0.834
C10	0.684	0.008	0.561–0.807
Combined amino acids and acylcarnitines			
Thr/C10:1	0.865	<0.001	0.782–0.947
Thr/C5-OH+C10:1+C18	0.839	<0.001	0.760–0.919
C5-OH+C10:1+C18	0.810	<0.001	0.723–0.896

ROC curve analysis of combined amino acids and acylcarnitines indicated that C5-OH+C10:1+C18, Thr/C10:1 and Thr/(C5-OH+C10:1+C18) all had an AUC >0.8 (0.810, 0.865, and 0.839, respectively, **Figure 2**). Among these indexes, C5-OH+C10:1+C18 <0.64 µmol/L and Thr/(C5-OH+C10:1+C18) >54 had the highest the specificity of 91.3% at the cost of reduced sensitivity (**Supplementary Table 5**). However, Thr/C10:1 >440 had good sensitivity and specificity at the same time (81.1 and 81.8%, respectively). The medium Thr/C10:1 level in the HH group was more than 3.5-times that in the KH group [893.2 (503.9, 1558.3) *vs* 249.0 (125.3, 428.8), *p* < 0.001]. When considering only patients who developed hypoglycemia during the fasting test, the sensitivity of Thr/C10:1 >440 was 80.8% (59 of 73 cases), and the specificity was 85.7% (12 of 14 cases). Because children in the KH group were older than children in the HH group, we further analyzed whether fasting Thr and C10:1 levels were associated with age by comparing them among children in the control group aged less than 1 year, from 1 year to 3 years and older than 3 years. Thr and C10:1 levels were not significantly different among the various age groups (*p* = 0.373 and *p* = 0.096). Therefore, Thr/C10:1 can be applied to children of all ages.

## Discussion

This study first described the spectra of amino acids and acylcarnitines in children with HH and KH in detail. The differences in amino acid and acylcarnitine levels at normoglycemia among HH, KH, and normal children suggest altered metabolic statuses caused by dysregulated secretion of insulin in HH.

Children with HH are in a state of chronic hyperinsulinemia. Thus, the metabolic statuses of lipid and amino acids might be different from those of normal children and children with KH. In the present study, the levels of short- (C4-OH, C5-OH), medium- (C8, C10, C10:1) and long-chain (C14:1, C18) acylcarnitines were significantly decreased in patients with HH compared to KH patients. Under normal circumstances, maintaining a normal blood glucose level through glycogenolysis can occur for only a limited time. Therefore, gluconeogenesis from non-carbohydrate compounds and ketogenesis act as alternative energy supply pathways for the body. Fat, the main energy source and main resource for ketogenesis, is mobilized in the form of fatty acids under the action of counter-regulatory hormones (25). Fatty acid oxidation is stimulated during hypoglycemia. Carnitine functions as a shuttle to bring long-chain fatty acids into the mitochondria for β-oxidation and then becomes acylcarnitine. Excess acylcarnitine is then released into the bloodstream (24). Thus, the acylcarnitine spectra can reflect fatty acid metabolic status and related disorders. Our study provided the first detailed evidence of acylcarnitine spectra profiles in infants and children with HH. Fatty acid mobilization was suppressed or maintained at a stable level in HH patients due to hypersecretion of insulin, resulting in relatively lower acylcarnitine levels in the HH group. Short- and medium-chain acylcarnitines also act as important energy sources, and enhanced short- and medium-chain fatty acid β-oxidation has been found in prematurity (26). Furthermore, except for fatty acid oxidation, short- and medium-chain acylcarnitine levels are also associated with other metabolic processes, including amino acid metabolism. The concentrations of some short-chain acylcarnitines were also related to the metabolism of BCAAs, which were the metabolic product of branched chain ketoacids (27). For example, an increase in C5-OH might be related to impaired catabolism of Leu when it is not ultimately converted into 3-methylglytaconyl-CoA but instead participates in another metabolic pathway to become C5-OH by crotonase and carnitine acyltransferase. This alternative pathway helps to save free CoA such that more CoA can undergo aerobic oxidation and facilitate the Krebs cycle (28). Interestingly, we found that C10:1 had the highest AUC for the prediction of HH among acylcarnitines. As a medium-chain acylcarnitine, the serum C10:1 level changed not only in patients with medium-chain acyl-CoA dehydrogenase deficiency but also in some non-metabolic diseases (12, 13, 29). Donovan et al. found that an increasing level of C10:1 was associated with an increasing number of wheezing episodes (12), and Xia et al. found that IgA nephropathy patients had a worse prognosis and treatment response if they had increased levels of C10:1 (13). However, the factors affecting changes in C10:1 and the specific role of C10:1 are still unclear, thus warranting more future studies.

As another important substrate for gluconeogenesis, the levels of amino acids in children with hypoglycemia were also changed. Excessive secretion of insulin inhibits proteolysis and stimulates amino acid uptake in the liver and muscle (30, 31). Therefore, children with HH exhibited hypoaminoacidemia compared to normal children, including reductions in Leu, Val, Pro, Arg, and Asp. The amino acid spectra of KH children were similar to those of normal children, except that KH children had relatively lower levels of Met, Thr, Arg, and Orn. Compared to HH children, KH children had lower levels of Thr, Met, Ala, and Glu but a higher level of Val; Haymond et al. also found lower Ala, Thr, Met and Orn levels during fasting compared with those of normal children. Most of these amino acids are gluconeogenic amino acids, which might be converted into ketone bodies more frequently in KH patients (32). Among these amino acids, Thr is the only amino acid that does not undergo deamination and transamination in metabolism but is directly catalyzed by threonine dehydratase, threonine dehydrogenase, and threonine aldolase, while other amino acids, including Thr, can be converted to butyryl coenzyme A, succinyl-coenzyme a, serine, glycine, etc. Excess Thr can increase the activity of lysine-α-keto gluconate reductase. Thus, the decreased Thr level in KH patients may be attributed to ketogenesis. However, the true mechanism requires further study in the future. Furthermore, the decreases in some amino acids in KH children might be a result of nutritional deficiencies because most of the 23 patients in the KH group experienced a recent decrease in intake or an increase in consumption. Thr and Met are essential amino acids and cannot be synthesized by the body. Felig et al. reported a decrease in amino acids, including Thr, Met, and Ala, after fasting in healthy adults, which may partly support our findings (33). Because insulin promotes the transport of BCAAs across cell membranes, BCAAs were decreased in the HH group (16). Chaussain et al. (16) also found a significant decrease in BCAAs in HH infants compared to KH infants and normal infants, which is consistent with our findings. However, we found a significant increase of Glu in the HH group compared to the other two groups. Glu plays an important role in signal transmission both extracellularly and intracellularly, participates in the regulation of insulin secretion, and can amplify glucose-stimulated insulin secretion (34). Mutations of insulin secretion-related pathways and different metabolic states may cause the increase in Glu in HH. Future studies will help us better understand the mechanism of amino acid and fat metabolism in children with HH.

The identification of focal lesions in HH is crucial for treatment selection for HH patients, and ^18^F-DOPA PET can precisely predict focal lesions in HH (19). Furthermore, focal lesions in HH are associated with specific genetic mutations (4). The spectra of amino acids and acylcarnitines were similar between HH children with focal lesions and those with diffuse lesions. These findings suggested that the changes in metabolic spectra are mainly caused by enhanced insulin secretion, and that the different types of pathogenesis may not have a significant impact on the metabolic spectra. Future studies should consider focusing on differences in metabolic profiles among HH patients with different genetic types.

Based on the above distinguishing metabolic features among the HH, KH, and normal groups, we found that Thr, C10:1 and Thr/C10:1 levels at normoglycemia might provide clues and aid in the diagnosis of HH, especially before the fasting test. At present, HH is diagnosed mainly based on serum indicators at the time of hypoglycemia, and blood samples must often be obtained through provocative fasting tests. Our findings offer new insights into the diagnosis of hypoglycemia in children prior to the fasting test, which may allow pediatricians to use more readily available samples to obtain an approximate idea of the type of hypoglycemia before the fasting test. Notably, however, insulin levels at the time of hypoglycemia remain the gold standard for diagnosing HH.

Because HH and KH are rare diseases, matching patients based on age was difficult. One of the limitations of our study was the age difference between the HH and KH groups. Patients in the HH group were 0.57 years younger than those in the control group and 1.46 years younger than those in the KH group due to the early onset of HH, which may cause potential age-matched metabolic profile changes. However, Van Rijt et al. compared the fasting spectra of acylcarnitines between children under 2 years of age and children between 2–7 years of age (35) and found no significant difference in their acylcarnitine profiles. Therefore, we believe that the age difference had only a limited effect on the current results. Furthermore, we noticed that some metabolites had relatively large scales, and overlaps between different groups were identified despite the lack of statistically significant differences among the groups. However, we still found a large difference between KH and HH in Thr and C10:1 levels.

In conclusion, our study confirmed the suppression of fatty acid mobilization and proteolysis caused by excessive secretion of insulin in HH children according to the detailed description of the spectra of amino acids and acylcarnitines at normoglycemia. However, the mechanisms that caused changes in certain amino acids and acylcarnitines are still largely unknown. Future studies are still needed to determine the detailed underlying mechanism of the above amino acid and acylcarnitine profile changes in HH.

## Data Availability Statement

The original contributions presented in the study are included in the supplementary material, and further inquiries can be directed to the corresponding author.

## Ethics Statement

The studies involving human participants were reviewed and approved by the Ethics Committee of Children’s Hospital of Fudan University (NO. 2015-154). Written informed consent to participate in this study was provided by the participants’ legal guardian/next of kin.

## Author Contributions

F-HL and M-YZ designed the study. Z-RX and X-YZ collected and analyzed the data. Z-RX wrote the first draft of the manuscript. F-HL and KH provided advice on the first draft and revised the manuscript. WL, W-HS, R-QC, M-YZ, J-WN, and LX helped with the clinical management of the patients. All authors contributed to the article and approved the submitted version.

## Funding

This project was funded by Shanghai Science and Technology Committee (15411961700).

## Conflict of Interest

The authors declare that the research was conducted in the absence of any commercial or financial relationships that could be construed as a potential conflict of interest.

## Abbreviations

HH, hyperinsulinemic hypoglycemia; KH, ketotic hypoglycemia; FFAs, free fatty acids; ROC, receiver operating characteristic; AUC, area under the curve; BCAAs, branched-chain amino acids. Abbreviations for amino acids and acylcarnitines are listed in **Appendix Table 1**.

## Appendix A

**Table A.1:** Abbreviations of amino acids and acylcarnitines.

Abbreviation	Full name
Ala	Alanine
Asp	Aspartate
Gly	Glycine
Leu	Leucine
Met	Methionine
Phe	Phenylalanine
Pro	Proline
Ser	Serine
Thr	Threonine
Trp	Tryptophane
Tyr	Tyrosine
Val	Valine
Arg	Arginine
Cit	Citrulline
Cr	Creatine
GAA	Guanidinoacetate
Glu	Glutamate
Orn	Ornithine
C0	Carnitine
C2	Acetylcarnitine
C3	Propionylcarnitine
C4	Butyrylcarnitine
C4-OH	Hydroxybutyrylcarnitine
C5	Isovalerylcarnitine/3-methylbutyrylcarnitine
C5-OH	3-Hydroxyisovaleryl carnitine/2-methyl-3-hydroxybutyrylcarnitine
C5:1	Penenylcarnitine
C6	Hexanoylcarnitine
C8	Octanoylcarnitine
C10	Decanoylcarnitine
C10:1	Decenoylcarnitine
C10:2	Decadienoylcarnitine
C12	Dodecanoylcarnitine
C14	Tetradecanoylcarnitine
C14-OH	Hydroxytetradecanoylcarnitine
C14:1	Tetradecenoylcarnitine
C16	Hexadecanoylcarnitine
C16-OH	Hydroxyhexadecanoylcarnitine
C16:1	Palmitoleylcarnitine
C18	Octadecanoylcarnitine
C18-OH	Hydroxyoctadecanoylcarnitine
C18:1	Octadecenoylcarnitine
