# Supplementary material for: Altered Serum Amino Acid and Acylcarnitine Profiles in Hyperinsulinemic Hypoglycemia and Ketotic Hypoglycemia
Source: Front Endocrinol (Lausanne). 2020 Oct 8;11:577373. doi: 10.3389/fendo.2020.577373 (PMC7579424; doi:10.3389/fendo.2020.577373)
Supplement: Supplementary file 1 [file DataSheet_1.docx]

Supplementary Material

**Supplemental Table 1** Normoglycemic amino acid concentrations of patients in the ketotic hypoglycemia, hyperinsulinemic hypoglycemia and control groups

| Amino acid^*^ | Ketotic hypoglycemia (n=23) | | Hyperinsulinemic hypoglycemia (n=74) | Control (n=170) | *P* value |
| --- | --- | --- | --- | --- | --- |
| Ala | 154.9 (117.0-219.6) | 229.9 (162.1-336.3) | | 192.1 (145.8-267.4) | 0.017^¶^ |
| Asp | 23.5 (17.7-28.1) | 21.6 (16.6-31.2) | | 26.5 (20.1-34.7) | 0.046^‡^ |
| Gly | 154.9 (122.3-178.1) | 179.2 (157.5-229.0) | | 170.0 (145.0-202.8) | 0.048 |
| Leu | 83.8 (72.3-117.6) | 82.8 (63.2-100.1) | | 91.3 (77.7-113.5) | 0.014^‡^ |
| Met | 15.4 (12.3-19.8) | 20.9 (16.8-28.3) | | 20.5 (16.7-27.1) | 0.005^†¶^ |
| Phe | 44.2 (35.5-58.1) | 47.5 (38.8-60.0) | | 45.5 (37.4-58.1) | 0.637 |
| Pro | 398.4 (287.0-564.4) | 367.1 (212.6-578.0) | | 448.9 (347.0-660.3) | 0.031^‡^ |
| Ser | 211.8 (105.6-299.3) | 182.9 (127.1-240.5) | | 182.0 (126.2-248.3) | 0.814 |
| Thr | 27.5 (20.5-34.0) | 42.2 (29.3-59.6) | | 35.8 (26.6-51.3) | 0.001^†¶^ |
| Trp | 9.6 (6.8-19.6) | 11.2 (7.8-16.1) | | 12.0 (8.6-18.4) | 0.416 |
| Tyr | 38.1 (31.6-53.0) | 48.4 (32.8-62.3) | | 50.1 (36.7-65.4) | 0.265 |
| Val | 109.5 (91.0-149.9) | 89.5 (63.2-115.7) | | 109.8 (90.6-135.3) | < 0.001^‡¶^ |
| Arg | 9.2 (6.2-15.9) | 11.0 (6.3-19.7) | | 16.9 (11.2-26.3) | < 0.001^†‡^ |
| Cit | 16.3 (11.9-21.2) | 15.7 (11.9-20.5) | | 17.7 (13.6-22.7) | 0.042 |
| Cr | 150.1 (107.5-212.8) | 153.4 (115.8-239.7) | | 162.2 (116.1-227.0) | 0.904 |
| GAA | 0.6 (0.3-1.0) | 0.5 (0.4-1.0) | | 0.5 (0.3-0.8) | 0.156 |
| Glu | 112.0 (95.2-144.1) | 168.3 (123.0-235.1) | | 138.3 (103.9-211.5) | 0.002^‡¶^ |
| Orn | 15.9 (12.5-21.5) | 19.9 (13.6-25.6) | | 21.9 (17.8-28.9) | 0.008^†^ |

*μmol/L, median (IQR). † *p* < 0.05 when the ketotic hyperinsulinemic hypoglycemia group *vs* the control group. ‡ *p* < 0.05 when the hyperinsulinemic hypoglycemia group *vs* the control group. ¶ *p* < 0.05 when the ketotic hyperinsulinemic hypoglycemia group *vs* the hyperinsulinemic hypoglycemia group.

**Supplemental Table 2** Normoglycemic amino acid concentrations of patients who developed hypoglycemia in the fasting test

| Amino acid^*^ | Ketotic hypoglycemia (n=14) | Hyperinsulinemic hypoglycemia (n=73) | *P* value |
| --- | --- | --- | --- |
| Ala | 157.8 (112.2-214.5) | 233.3 (162.8-339.2) | 0.012 |
| Asp | 23.5 (17.7-28.1) | 21.6 (16.6-31.2) | 0.682 |
| Gly | 165.5 (122.5-177.2) | 177.7 (157.5-231.0) | 0.053 |
| Leu | 79.7 (71.4-99.6) | 83.4 (63.7-100.6) | 0.665 |
| Met | 15.3 (12.3-17.8) | 20.9 (16.8-28.4) | 0.004 |
| Phe | 43.0 (39.2-57.8) | 48.6 (39.3-60.1) | 0.413 |
| Pro | 398.4 (287.0-564.4) | 367.1 (212.6-578.0) | 0.894 |
| Ser | 211.8 (105.6-299.3) | 182.9 (127.1-240.5) | 0.206 |
| Thr | 27.5 (20.5-34.0) | 42.2 (29.3-59.6) | < 0.001 |
| Trp | 9.6 (6.8-19.6) | 11.2 (7.8-16.1) | 0.177 |
| Tyr | 38.9 (31.0-42.9) | 48.9 (33.1-62.4) | 0.084 |
| Val | 109.5 (91.0-149.9) | 89.5 (63.2-115.7) | 0.040 |
| Arg | 10.5 (8.1-15.5) | 11.1 (6.6-19.7) | 0.959 |
| Cit | 16.6 (12.7-20.7) | 15.8 (11.9-20.5) | 0.699 |
| Cr | 154.2 (134.9-211.4) | 155.5 (115.8-240.4) | 0.716 |
| GAA | 0.6 (0.4-1.0) | 0.5 (0.4-1.0) | 0.768 |
| Glu | 107.1 (95.1-141.5) | 168.3 (122.0-235.8) | 0.001 |
| Orn | 15.8 (10.7-21.1) | 19.8 (13.6-25.4) | 0.227 |

*μmol/L, median (IQR).

**Supplemental Table 3** Normoglycemic acylcarnitine concentrations of patients in the ketotic hypoglycemia, hyperinsulinemic hypoglycemia and control groups

| Acylcarnitine^*^ | Ketotic hypoglycemia (n=23) | Hyperinsulinemic hypoglycemia (n=74) | Control (n=170) | *P* value |
| --- | --- | --- | --- | --- |
| C0 | 25.14 (20.88-28.89) | 32.98 (24.76-40.10) | 30.62 (24.07-36.70) | 0.122 |
| C2 | 9.87 (6.97-10.50) | 13.18 (9.51-19.17) | 12.49 (9.63-16.89) | 0.175 |
| C3 | 1.02 (0.79-1.16) | 1.17 (0.90-1.55) | 1.23 (0.79-1.61) | 0.386 |
| C4 | 0.14 (0.11-0.18) | 0.13 (0.10-0.13) | 0.15 (0.12-0.19) | 0.007^†^ |
| C4-OH | 0.08 (0.05-0.13) | 0.05 (0.04-0.08) | 0.07 (0.05-0.13) | < 0.001^†‡^ |
| C5 | 0.07 (0.06-0.08) | 0.07 (0.05-0.09) | 0.08 (0.06-0.10) | 0.065 |
| C5-OH | 0.18 (0.14-0.19) | 0.12 (0.09-0.15) | 0.14 (0.10-0.18) | < 0.001^†‡^ |
| C5:1 | 0.03 (0.01-0.04) | 0.02 (0.01-0.03) | 0.02 (0.01-0.04) | 0.309 |
| C6 | 0.04 (0.03-0.05) | 0.04 (0.03-0.05) | 0.05 (0.03-0.06) | 0.002^†^ |
| C8 | 0.05 (0.04-0.06) | 0.04 (0.03-0.05) | 0.06 (0.04-0.09) | < 0.001^†‡^ |
| C10 | 0.09 (0.06-0.10) | 0.07 (0.04-0.10) | 0.09 (0.06-0.14) | < 0.001^†‡^ |
| C10:1 | 0.10 (0.05-0.12) | 0.04 (0.03-0.07) | 0.08 (0.05-0.12) | < 0.001^†‡^ |
| C10:2 | 0.02 (0.01-0.02) | 0.01 (0.01-0.02) | 0.02 (0.01-0.03) | 0.002^†^ |
| C12 | 0.06 (0.04-0.07) | 0.06 (0.04-0.09) | 0.08 (0.05-0.11) | 0.008^†^ |
| C14 | 0.07 (0.06-0.09) | 0.10 (0.08-0.14) | 0.11 (0.07-0.14) | 0.398 |
| C14:1 | 0.05 (0.03-0.10) | 0.03 (0.02-0.05) | 0.05 (0.03-0.07) | < 0.001^†‡^ |
| C16 | 0.92 (0.80-1.04) | 0.97 (0.78-1.41) | 0.89 (0.72-1.12) | 0.218 |
| C16:1 | 0.05 (0.04-0.06) | 0.05 (0.04-0.07) | 0.05 (0.04-0.07) | 0.513 |
| C18 | 0.46 (0.40-0.51) | 0.35 (0.26-0.43) | 0.40 (0.32-0.51) | < 0.001^†‡^ |
| C18:1 | 0.76 (0.65-0.95) | 0.77 (0.51-0.89) | 0.73 (0.52-0.93) | 0.190 |

*μmol/L, median (IQR). † *p* < 0.05 when the ketotic hyperinsulinemic hypoglycemia group *vs* the control group. ‡ *p* < 0.05 when the hyperinsulinemic hypoglycemia group *vs* the control group. ¶ *p* < 0.05 when the ketotic hyperinsulinemic hypoglycemia group *vs* the hyperinsulinemic hypoglycemia group.

**Supplemental Table 4** Normoglycemic acylcarnitine concentrations of patients who developed hypoglycemia in the fasting test

| Acylcarnitine^*^ | Ketotic hypoglycemia (n=14) | Hyperinsulinemic hypoglycemia (n=73) | *P* value |
| --- | --- | --- | --- |
| C0 | 23.50 (19.57-25.63) | 32.65 (24.61-40.41) | 0.049 |
| C2 | 9.87 (6.52-10.53) | 13.23 (9.74-19.20) | 0.085 |
| C3 | 0.95 (0.77-1.07) | 1.19 (0.92-1.55) | 0.853 |
| C4 | 0.14 (0.11-0.18) | 0.12 (0.10-0.19) | 0.300 |
| C4-OH | 0.08 (0.06-0.13) | 0.05 (0.04-0.08) | 0.017 |
| C5 | 0.07 (0.06-0.08) | 0.07 (0.05-0.09) | 0.991 |
| C5-OH | 0.18 (0.13-0.19) | 0.12 (0.09-0.15) | 0.001 |
| C5:1 | 0.03 (0.01-0.04) | 0.02 (0.01-0.03) | 0.206 |
| C6 | 0.03 (0.03-0.05) | 0.04 (0.03-0.05) | 0.470 |
| C8 | 0.05 (0.05-0.06) | 0.04 (0.03-0.06) | 0.013 |
| C10 | 0.09 (0.07-0.10) | 0.07 (0.05-0.10) | 0.053 |
| C10:1 | 0.10 (0.05-0.13) | 0.04 (0.03-0.07) | 0.001 |
| C10:2 | 0.02 (0.01-0.03) | 0.01 (0.01-0.02) | 0.040 |
| C12 | 0.06 (0.04-0.07) | 0.06 (0.04-0.09) | 0.380 |
| C14 | 0.06 (0.06-0.08) | 0.10 (0.08-0.14) | 0.140 |
| C14:1 | 0.07 (0.03-0.10) | 0.03 (0.02-0.05) | 0.005 |
| C16 | 0.93 (0.78-1.05) | 0.97 (0.78-1.42) | 0.813 |
| C16:1 | 0.05 (0.04-0.07) | 0.05 (0.04-0.07) | 0426 |
| C18 | 0.43 (0.40-0.50) | 0.35 (0.27-0.43) | 0.008 |
| C18:1 | 0.75 (0.61-0.96) | 0.78 (0.53-0.90) | 0.358 |

*μmol/L, median (IQR).

**Supplemental Table** **5** Sensitivity and specificity of biomarkers

| Criteria | Sensitivity^a^ | Specificity^a^ | Sensitivity^b^ | Specificity^b^ |
| --- | --- | --- | --- | --- |
| Insulin > 1 mIU/L |  |  | 93.2% | 42.9% |
| Insulin > 2 mIU/L |  |  | 90.4% | 64.3% |
| C10:1 < 0.09 μmol/L | 81.1% | 72.7% | 80.8% | 71.4% |
| Thr > 35μmol/L | 66.2% | 78.3% | 65.8% | 85.7% |
| Thr/C10:1 > 440 | 81.1% | 81.8% | 80.8% | 85.7% |
| Thr/(C5-OH+C10:1+C18) > 54 | 70.3% | 91.3% | 69.9% | 92.6% |
| C5-OH+C10:1+C18 < 0.64 μmol/L | 70.3% | 91.3% | 69.9% | 85.7% |

a: calculated among all cases (74 cases of HH and 23 cases of KH). b: calculated among those who developed hypoglycemia in the fasting test (73 cases of HH and 14 cases of KH).

121 patients with hypoglycemia

24 cases excluded:

-Glycogen storage diseases (n=14)

-Pituitary dysplasia (n=5)

-Adrenal disorders (n=2)

-Organic acid metabolism disorders (n=3)

74 cases of HH and 23 cases of KH

Fasting test

(Insulin and FFA levels were tested at the end of the fasting test)

^18^F DOPA PET

(50 cases of HH)^a^

Spectra of amino acid and acylcarnitine

(blood sample at normoglycaemic status)

5 cases who denied to take the fasting test were excluded (5 cases of KH)

Focal lesions: 17 cases

Diffuse lesions: 33 cases

74 cases of HH and 18 cases of KH

5 cases failed to develop hypoglycemia within 12 hours were excluded (1 case of HH and 4 cases of KH)

74 cases of HH and 23 cases of KH^ac^

87 cases developed hypoglycemia (73 cases of HH and 14 cases of KH)^abc^

**Supplemental Figure 1.** Subjects recruited flow chart

a: compare the spectra of amino acid and acylcarnitine between KH an HH. b: calculate the sensitivity and specificity of insulin. c: calculate the sensitivity and specificity of amino acids and acylcarnitine.
